# Hygienic Aphorisms

**Published:** 1882-01

**Authors:** Felix L. Oswald


### SOME HYGIENIC APHORISMS.

THE first thing a child should learn is to ask for a drink of water. I have seen hand-fed children scream and fidget for hours together, as if troubled by some unsatisfied want, but at the same time rejecting the milk-bottle and pap-dish with growing impatience. In nine such cases out of ten the nurse will either resort to paregoric or try the effect of a lullaby. I need not say that the poison expedient would be wrong under any circumstances, but, before you try anything else, offer the child a cup of cold water. To a young nursling, the mother's breast supplies both food and drink, but farinaceous paps require a better diluent than milk.

If I should name the greatest danger of childhood, I would unhesitatingly say—medicine. A drastic drug as a remedial agent is Beelzebub in the *role* of an exorcist.

Tight-swaddling, strait-jacket gowns and trailing petticoats—restraint, in short, makes our infants peevish. If we would give them a chance to use their limbs they would have no time to scream.

It would prevent innumerable diseases if people would learn to distinguish a morbid appetency from a healthy appetite. One diagnostic rule is this: that the gratification of the latter is not followed by repentance. Another, that the former has to be artificially and painfully acquired; our better nature resists the incipience of a morbid "second nature." After acquitting Nature from all responsibility for such factitious appetites, it may be justly said that a man can find a road to health and happiness by simply following his instincts.

The supposed danger of cold drinks on a hot day is a very expensive superstition. It deprives thousands of the most pleasurable sensation the human palate is capable of. It is worth a two hours' *anabasis* in the dog-days to drink your fill at the coldest rock-spring of the mountains.

Bathing in flannel! I would as soon take ice-cream in capsules. The price of the flannel suit would buy you a season ticket to some lonely beach.

"A catarrh is the beginning of a lung-disease." It would be the end of it, if we did not aggravate it with nostrums and fusty sickrooms.

Somehow or other we must have abused our teeth shamefully before Nature had to resort to such a veto as a toothache. A tooth pulled in time saves nine.

"If you doubt whether a contemplated act is right or wrong," says Zoroaster, "it is the safest plan to omit it." Let dyspeptics remember that when they hesitate at the brink of another plateful.

The digestion of superfluous food monopolizes the vital energy; hence, the mental and physical indolence of great eaters. Strong-headed business-men manage to conquer that indolence, but only by an effort that would have made the fortune of a temperate eater.

A glutton will find it easier to reduce the number of his meals than the number of his dishes.

Highland children are the healthiest, and, even starving, the happiest. "There is no joy the town can give like those it takes away."

Paracelsus informs us that the composition of his "triple panacea" can be described only in the language of alchemistic adepts. Nature's triple panacea is less indescribable—fasting, fresh air and exercise.

A banquet without fruit is a garden without flowers.

"Do animals ever go to the gymnasium?" asks an opponent of the movement cure. Never; they have no time; they are too busy practicing gymnastics out-doors.

Descent from a long-lived race is not always a guarantee of longevity. A far more important point is the sanitary condition of the parents at the birth of the child. Pluck, however, is hereditary, and has certainly a prophylactic, "health-compelling" influence.

The first gray hairs are generally a sign of *dear-bought* wisdom.

The "breaking up" of a pulmonary disease could often be accomplished by *breaking* the bed-room windows.

Death, formerly the end of health, is nowadays the end of a disease. Dying a natural death is one of the lost arts. [Which pure Homœopathy restores.—Ed.]

There seems to be a strange *fatum* in the association of astronomy with humbug: formerly in horoscopes, and now in patent-medicine almanacs. A patent-medicine man is generally the patentee of a device for selling whiskey under a new name.

A "chronic disease," properly speaking, is nothing but nature's protest against a chronic provocation. To say that chronic complaints end only with death, means, in fact, that there is generally no other cure for our vices.

Every night labors to undo the physiological mischief of the preceding day—at what expense gluttons may compute if they compare the golden dreams of their childhood with the leaden torpor-slumbers of their pork and lager-beer years.

If it were not for calorific food and superfluous garments, midsummer would be the most pleasant time of the year.—Dr. FELIX L. OSWALD in *Popular Science Monthly*.

